# Modified Bilhaut–Cloquet procedure for Wassel type III–IV polydactyly

**DOI:** 10.1186/s13018-024-04553-x

**Published:** 2024-01-16

**Authors:** Xuzhao Guo, Yanfei Wei, Fei Wang, Xiaokang Zhou, Shuo Zhao

**Affiliations:** 1 Department of Orthopaedics, Children’s Hospital of Hebei, Shijiazhuang City, Hebei Province People’s Republic of China; 2 Department of Surgery 1, Zanhuang County Hospital, Shijiazhuang City, Hebei Province People’s Republic of China

**Keywords:** Polydactyly, Modified Bilhaut–Cloquet, Surgery

## Abstract

**Background:**

To investigate the functional and aesthetic results of a new modified Bilhaut–Cloquet procedure for the treatment of Wassel type III–IV thumb polydactyly.

**Methods:**

Thirteen patients with Wassel type III–IV thumb polydactyly who visited the Department of Orthopedics of Hebei Provincial Children's Hospital from 2019 to 2022 were selected. The surgical procedure involved a modified Bilhaut–Cloquet surgery, where two-thirds of the distal part of the dominant finger was retained as the p body of the reconstructed thumb. The triangular bone block of the ablated distal thumb that did not contain the epiphysis and articular cartilage was sutured and fixed, and the neurovascular flap of the ablated distal thumb was used as an augmenting segment of the reconstructed thumb, with the nail bed and nail matrix exquisitely sutured. The evaluation performed according to the Japanese Society for Surgery of the Hand (JSSH) system.

**Results:**

All 13 children showed bone healing, no wound infection, nonunion, or deformity healing. None of the children showed a significant reduction in the active and passive mobility of the thumb postoperatively compared with preoperatively. Postoperative evaluation was performed based on the JSSH score, with a mean of 17.15 points (14–19 points), with 11 children rated as excellent and two as good. No severe nail ridges, nail gaps, or nail split deformities of the thumb were observed postoperatively. Postoperative metacarpophalangeal and interphalangeal joint movements were not reduced compared with preoperative movements. All parents were satisfied with the appearance and function of the reconstructed thumb.

**Conclusion:**

The modified Bilhaut–Cloquet procedure designed in this study was satisfactory for Wassel type III–IV thumb polydactyly without affecting the stability of the interphalangeal joints and preserving joint mobility. The postoperative thumb has a comparable circumference and nail width and was cosmetically and functionally satisfactory, especially for the asymmetric two thumbs, which achieved favorable results.

## Introduction

### Background

Congenital polydactyly of the thumb is the most common congenital deformity of the hand in newborns, resulting from abnormal differentiation of limb buds at 6–8 weeks of the embryonic period. The reported incidence is approximately 0.08–1‰ [1]. Treatment is surgery-based techniques, such as simple resection, reconstruction following the resection [2–4], and the Bilhaut–Cloquet procedure [5]. Simple resection is indicated for most Wassel I, III, and V polydactyly cases. For Wassel II, IV, and VI polydactyly at the level of the articular and epiphyseal joints, resection followed by articular, bony, and tendon reconstruction resolves most of the problems. In rare cases of Wassel types III and IV, such as those in which both digits in the thumb are hypoplastic and narrow, the conventional Bilhaut–Cloquet procedure aims to achieve a more satisfactory thumb appearance than reconstruction in terms of nail width, finger circumference, and interphalangeal joint stabilization with favorable alignment postoperatively. However, the Bilhaut–Cloquet procedure has significant drawbacks, such as nail deformity, impaired digit growth, and limited joint mobility, among other complications. Modifications to the Bilhaut–Cloquet procedure substantially alleviated the severity of complications [6–10]. The Bilhaut–Cloquet procedure has been gaining acceptance among surgeons and parents. To address the impaired growth and joint mobility of the digits after Bilhaut–Cloquet surgery, we designed a new modified approach based on the traditional Bilhaut–Cloquet procedure that does not involve maneuvering of the epiphyses and joints, thus minimizing complications such as impaired growth and limited joint mobility. In this study, we performed a modified Bilhaut–Cloquet procedure to treat the type III and type IV polydactyly and reviewed and analyzed the initial follow-up results.

## Materials and methods

### Patient study

This retrospective study was conducted at the Department (for peer review) between January 2019 and August 2022. The study was approved by the Ethics Committee (for peer review). The inclusion criteria were as follows: Wassel types III and IV polydactyly of the thumb; the circumference of each thumb was < 70% of the contralateral thumb, and the nail width was < 60%. The exclusion criteria were thumb circumference was > 70% of the contralateral thumb and nail width was > 60% of the contralateral thumb. All patients underwent anteroposterior radiography to determine the Wassel classification joint alignment and angulation deformity. The mean patient age was 14 months (range: 11–26 months) (Table 1). There were six male and seven female patients; regarding polydactyly, eight patients were right-sided, five left-sided, twelve cases were unilateral, and one was bilateral.

**Table 1:** Patients data

Patient	Gender	Age (months)	Classification	Affect site	Follow time (months)
1	Male	11	III	Right	30
2	Female	14	IV	Left	18
3	Female	13	IV	Left	20
4	Female	12	IV	Right	24
5	Female	16	IV	Bilateral	22
6	Male	12	IV	Left	24
7	Male	11	IV	Left	13
8	Female	18	IV	Right	19
9	Male	11	IV	Right	18
10	Female	12	IV	Left	20
11	Female	14	III	Left	24
12	Female	13	III	Right	30
13	Male	26	IV	Left	38

### Surgery technique

**Fig. 1:**
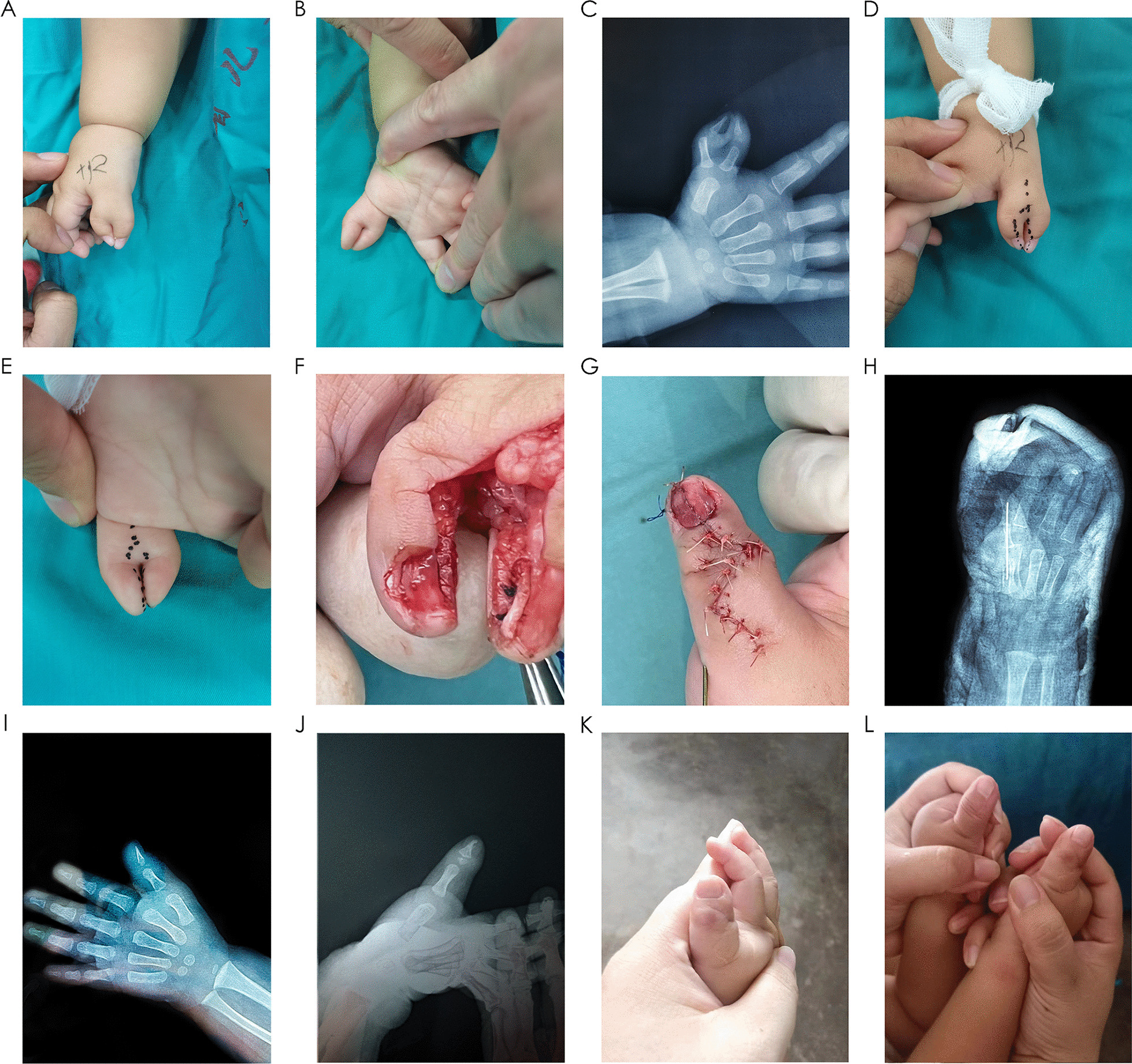
Case 5. **A** and **B** Preoperative of image. **C** Preoperative x-ray. **D** and **E** Draw incision. **F** Bone and nail assembly in surgery. **G** Immediate postoperative appearance. **H** Postoperative X-ray. **I** X-ray of 4 weeks after operation (K-wire removed). **J** Postoperative X-ray at 22 months. **K** and **L** Appearance following modified Bilhaut–Cloquet procedure at 22 months

**Fig. 2:**
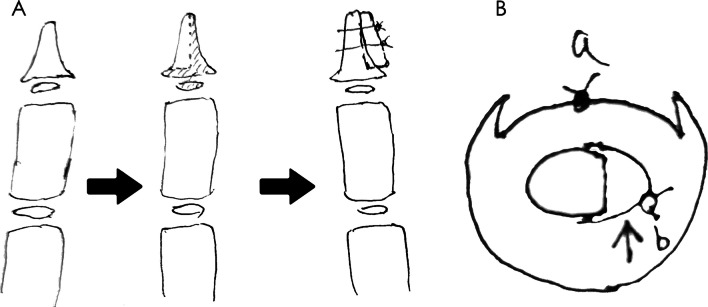
**A** The modified Bilhaut–Cloquet procedure for a Wassel type-IV bifid thumb. The shadow area is resected then the reserved distal phalangeal bone combined by PDS-II stitching. **B** the schematic drawing of the technique, arrow: the reserved section of resected thumb, a: the suture plane of the nail bed, b: Sutures are knotted and secured on the ventral side to restore the curvature of the phalanges. The fusion plane of the distal phalangeal bone is not in the same plane as the suture plane of the nail bed

Before surgery, the dominant and the resection thumbs were determined; the thumb with stable metacarpophalangeal and interphalangeal joints, better flexion and extension activities were regarded as the dominant thumb (12 on the ulnar side and one on the radical side). The circumference of the reconstructed thumb and the nail width were determined based on the contralateral thumb. The dominant thumb underwent a longitudinal incision halfway through the nail, extending in a Z-shape proximally from the longitudinal incision. One-third of the radial or ulnar side was resected to remove the nail bed, matrix, and nail fold, preserving the remaining two-thirds (Fig. 1D–E). A longitudinal incision was made on the ablated thumb, determined by the nail width on the contralateral side. The triangular bone block, nail bed, matrix, and soft-tissue flap from the ablated thumb were removed. The trimmed triangular bone block reserved from the ablated thumb had its epiphysis and articular cartilage removed, retaining the cortical and cancellous bones. The trimmed triangular bone block was attached to the radial or ulnar side of the distal phalanx of the preserved thumb, the periosteum was sliced to expose the cortex, and a hole was punched through it transversely to encircle the triangular bone block around the radial or ulnar side of the distal phalanx to structure the reconstructed thumb. The bone block was fixed from the radial or ulnar side of the ablated thumb, and the dominant thumb phalanx was aligned with the bone tunnel using 5–0 PDS-II sutures (ensuring no gap existed) to confirm that the fusion surface was flat (Fig. 1F). The bone union surface was smooth (Fig. 1H). As the triangular bone block had the natural sagittal curvature of the phalanx, the reconstruction mirrored the natural shape of the phalanx after splicing (Fig. 2). The nail bed, lunula, and matrix were microscopically aligned flat with an 8–0 thread and the suture was not too tight to avoid the formation of a visible bulge that could lead to nail ridge deformity in the distant future. The flap was sutured and trimmed until the thumb circumference was approximately the same as that of the contralateral side. The nail plate was replanted to cover the nail bed, facilitate healing, and reduce bleeding after suturing. In cases where the interphalangeal joint exhibits an oblique and articular surface exceeding 20°, the proximal phalanx should first undergo closed wedge osteotomy orthopedic internal fixation. Postoperatively, the thumb was fixed with a cast in the opposite position.

### Aftercare

After removing the Kirschner's pin, functional exercises were performed in all patients after 4–6 weeks, depending on bone healing. Postoperative evaluations were performed at 6 months, 1 year, and 2 years according to the JSSH score.

## Results

The mean follow-up period was 23 months (range, 13–38 months). The mean age of the patients at the time of surgery was 14 months (range, 11–26 months). All 13 children showed bone healing, no wound infection, bone nonhealing, or deformity healing. Compared with the preoperative values, postoperative active and passive thumb mobility in all children was not different after our designed modified Bilhaut–Cloquet procedure (Table 2). Postoperative assessment based on the JSSH score was excellent in 11 children and good in two (Table 3). The reconstructed thumbs were larger than the contralateral side in all 13 children postoperatively, which was favorable for gripping, pinching and opposition, but appeared bulky. The nail had no serious nail ridge or split deformity and its width, curvature, and appearance were similar to those of the contralateral thumb. All parents were satisfied with the appearance and function of the reconstructed thumb.

**Table 2:** Patient data

Patient	ROM of MCP (pre)	ROM of MCP (post)	ROM of IP (pre)	ROM of IP (post)	Align of MCP (post)	Align of IP (post)	Postoperative Nail Width Ratio*	Postoperative circumstance Ratio*	Ridging of Nail	Seagull deformity
1	50	50	30	25	8	0	1.05	1.2	Absent	Absent
2	45	50	25	20	15	5	1	1.1	Absent	Absent
3	50	50	25	15	10	5	1.15	1.25	Absent	Absent
4	55	50	30	25	15	5	1	1.1	Minor	Absent
5	60	60	35	30	5	0	1.1	1.2	Absent	Absent
6	60	55	30	30	15	5	1.05	1.1	Minor	Absent
7	65	60	35	25	10	5	1	1.1	Absent	Absent
8	60	55	15	10	5	0	1.1	1.3	Minor	Absent
9	45	40	10	10	15	15	1.1	1.3	Absent	Absent
10	55	50	25	15	8	5	1.05	1.35	Minor	Absent
11	50	40	30	20	10	10	1.05	1.2	Absent	Absent
12	60	50	25	15	15	10	1	1.15	Absent	Absent
13	40	35	15	10	25	15	1.1	1.4	Minor	Absent

*Affected/contralateral

**Table 3:** JSSH scores of patient

Patient	Abnormal alignment of IP (0–2)	Abnormal alignment of MCP (0–2)	Instability of IP (0–2)	Instability of MCP (0–2)	Active motion (Flexion/ palmar abduction) (0–4)	Extension Lag (0–2)	Appearance (0–4)	Subjective (0–2)	JSSH
1	2	2	1	2	3	2	4	2	18
2	2	1	2	2	3	1	4	2	17
3	2	2	2	2	3	1	4	2	18
4	2	1	2	2	3	2	3	2	17
5	2	2	2	2	4	2	3	2	19
6	2	1	2	2	3	1	4	2	17
7	2	2	2	2	3	1	4	2	19
8	2	2	2	2	3	1	4	2	18
9	1	1	1	2	2	2	4	2	15
10	2	2	2	2	3	2	3	2	18
11	1	2	2	2	3	1	4	2	17
12	1	1	2	2	3	2	4	2	17
13	1	1	2	1	2	1	3	2	14

## Discussion

In cases of Wassel III and IV polydactyly, in which both thumbs are hypoplastic, simple excision is unacceptable to parents because of the slenderness of the thumb and narrowness of the nail. Soft tissue reconstruction of the thumb after resection also fails to provide a perfectly reconstructed thumb appearance, presenting with a large circumference and narrow nail, and there may be long-term complications, such as radial deviation or Z deformity. For patients with type III–IV polydactyly with angulation > 20°, thumb resection followed by osteotomy to correct angulation, flexor and extensor tendon centralization, and soft tissue augmentation can provide desirable circumference, appearance, joint stability, and alignment. However, esthetics pose a primary challenge in situations where both thumbs are skinny and severe nail narrowing is present.

Initially, a procedure designed by Bilhaut–Cloquet in 1889, it achieved satisfactory thumb circumferences, nail widths, and alignment in polydactyly with hypoplasia of both thumbs and improved appearance and function. However, owing to the stitching of the epiphyses and articular surfaces, even with careful alignment, there may still be significant developmental impairment, growth inconsistency, inevitable joint stiffness limited mobility, and esthetic deficiencies of the nail. With advances in anesthesia, most cases of polydactyly can be performed at a younger age (up to 1 year). In younger infants, finger dysplasia and small bones require more delicate surgical maneuvers, and the epiphyseal and articular surfaces, even when tightly closed under direct visualization, may have complications such as epiphyseal damage and other inconsistencies. Most of the epiphyses of the thumb in this age group are within 1 mm in width, and what might seem to be "anatomical" repositioning to the naked eye could still indicate a subtle misalignment in actuality. Detecting this mismatch is difficult with the naked eye; however, it gradually magnifies with growth, resulting in growth inconsistencies.

With the advancement of microsurgical sutures and techniques, fine alignment of the nail bed and nail matrix has become possible, and seagull and severe nail split deformities have gradually decreased. Iwasawa designed a modified Bilhaut–Cloquet procedure with floating bone block filling to restore the curvature of the nail bed and fine alignment of the nail bed and lunula in cases of hypoplastic polydactyly with asymmetric nails; the results of the procedure at an average of 12 years of follow-up showed a satisfactory nail appearance and mild nail deformity [11]. Baek designed the splicing of the terminal phalangeal bone block outside the articular surface and axial rotation to restore the natural curve without affecting the epiphysis and articular surface, resulting in a high postoperative joint mobility, without seagull deformity following the operation [12]. Regarding Samson's modified design of the Bilhaut–Cloquet procedure for suturing the bone, skin, and nails without suturing the nail bed and matrix, the postoperative appearance was good, with a single case of the longitudinal ridge of the nail [8]. Our technique aimed to widen the distal phalanx laterally using a triangular bone block, which involved passing a transverse Kirschner's needle through the bone tunnel followed by PDS line suture to pressurize and fix the block. Because of the roundness of the palmar curvature of the distal phalanx, the sutures were knotted on the palmar side after fixation. Consequently, the final curvature of the reconstructed distal phalanx appeared natural, and the width increased significantly. Microscopic closure of the nail bed, matrix, and eponychium was achieved using 8–0 sutures, retaining a slight tension. Eventually, after the bone heals, it becomes less prone to collapse and bone cleft, leading to insufficient support of the nail bed in the long term and the appearance of nail ridges or seagull deformities. Abid offers a modified Bilhaut–Cloquet procedure for the repair of type IV-D thumb polydactyly, in which a wedge-shaped osteotomy of the base of the distal phalanx of one thumb is joined to a triangular base bone block of the other thumb that contains the lateral collateral ligaments and articular capsule. The middle phalanx is spliced according to the conventional Bilhaut–Cloquet procedure, with the nail bed left undisturbed. Postoperatively, the alignment was good, and growth was not affected; however, joint stiffness and mobility were poor [13]. The modified Bilhaut–Cloquet used in this study did not join the epiphysis and articular cartilage and avoided growth impairment or joint mismatch. This study was designed to perform bony procedures outside the joint because regardless of how carefully the articular cartilage and its attached structures, such as the joint capsule and lateral collateral ligaments, were handled, the original joint mobility and stability could not be preserved and were lost. The design preserved the range of motion and joint stability of the thumb without aggravating the limitations of motion and joint stiffness (*p* > 0.05). Although active postoperative rehabilitation can help improve joint stiffness and mobility, the exercise of infants and young children depends entirely on the parents, which makes it challenging to ensure compliance, which is especially important during preoperative communication and counseling, as well as postoperative follow-up. Maillet reviewed 10 patients who underwent Bilhaut–Cloquet surgery and had a 10° reduction in metacarpophalangeal joint mobility and a 30° reduction in interphalangeal joint mobility postoperatively but had satisfactory function [14]. Hand surgeons generally agree that the primary function of the thumb is opposition, followed by abduction, flexion, and extension. Even when the metacarpophalangeal and interphalangeal joints are considerably restricted, they typically do not affect a child's daily activities. Therefore, when a child's routine remains unaffected, parents often find actively engaging the child in rehabilitation exercises challenging. This difficulty may also contribute to postoperative joint stiffness and reduced mobility.

The main indications for the traditional Bilhaut–Cloquet procedure are that both thumbs should have approximately the same length, circumference, and width of the epiphysis to minimize the complications of growth restriction. The incision designed at the dorsal and abdominal medials of the thumb affects esthetics and pain and interferes with tactile sensation. Bo He's modified Bilhaut–Cloquet procedure, which has a dorsal ulnar aspect of the incision, improves medial scarring of the traditional Bilhaut–Cloquet procedure, and the incision is designed in the dorsal part of the thumb. No severe deformities of the nail have been observed [15]. The modified Bilhaut–Cloquet procedure designed in this study did not involve the epiphysis and accessory structures, such as the articular surface and joint capsule, preserved the stability and integrity of the distal thumb and interphalangeal joints, used the relatively well-developed ulnar thumb as the preserved thumb, and utilized the flap of the resected thumb and the triangular-shaped bone block to widen and thicken the thumb. Thus, the appearance and function of the reconstructed thumb can be closer to the contralateral side without affecting its function. The bone splice and nail bed suture planes were no longer in the same sagittal plane, thus effectively avoiding bone plane unevenness secondary to nail deformity and more effectively restoring the natural curve of the nail. The nail bed and matrix were repaired using an 8–0 thread under a microscopic, low-tension suture to minimize the occurrence of nail ridge, nail split, and seagull deformity. In cases with eccentricity of the flexor and extensor tendons and angulation of the articular surfaces, closed-wedge osteotomy of the proximal phalanx and centering of the abnormal flexor and extensor tendons should be performed to improve alignment. Ezaki [16] compared the characteristics of the Tada [17], Cheng [18], and JSSH [19] assessment systems and concluded that the JSSH assessment system had improved reliability and that the overall result categories were more comprehensive than those of the other methods. In this study, 11 cases were excellent, and the other two were good after surgery, according to the JSSH assessment. The curvature and width of the nails of 13 children were close to the contralateral thumb, and there were no severe nail ridges, nail splits, or seagull deformities. Following the surgery, the appearance and function of the thumbs satisfied the parents of the children.

The modified Bilhaut–Cloquet procedure in this study is recommended as a supplemental step for the distal part of the thumb. It utilizes the advantageous parts of both thumbs to the fullest extent and obtains good function and appearance of the reconstructed thumb with a relatively simple, non-harassing operation on the epiphysis and joint. This method could be applied to all types of polydactyly.

### Disadvantages

This retrospective study had a relatively short follow-up time and required further medium- and long-term follow-ups to assess this effect. No random and control groups were included. The number of cases was small, and expanding the number of patients will enhance the credibility of the findings.

## Conclusions

The modified Bilhaut–Cloquet procedure designed in this study was satisfactory for Wassel type III–IV thumb polydactyly without affecting the stability of the interphalangeal joints and preserving joint mobility. The postoperative thumb had a comparable circumference and nail width and was cosmetically and functionally satisfactory, especially for the two asymmetric thumbs, which achieved favorable outcomes.
